# Kidney Transplant Outcomes With Non-Depleting Antibody Induction Therapy in Human Leucocyte Antigen Sensitised Recipients

**DOI:** 10.3389/ti.2025.14852

**Published:** 2025-09-30

**Authors:** Ria Nagpal, Katie Butler, Nicola Thal, Abigail Hobill, Alice Gage, Maryam Javed, Felix Karst, Azhar Ali Khan, Amy Needleman, Graham Shirling, Henry Stephens, Sharon Vivers, Franco Tavarozzi, Neema Mayor, Sandra Frater, Alan Salama, Mark Harber, Gareth Jones, Raymond Fernando, Rhys D. R. Evans

**Affiliations:** ^1^ Department of Renal Medicine and Transplantation, Royal Free Hospital NHS Foundation Trust, London, United Kingdom; ^2^ Anthony Nolan Histocompatibility Laboratories, London, United Kingdom; ^3^ UCL Centre for Kidney and Bladder Health, Royal Free Hospital, London, United Kingdom; ^4^ Anthony Nolan Research Institute, London, United Kingdom; ^5^ UCL Cancer Institute, London, United Kingdom

**Keywords:** rejection, survival, Epitopes, HLA allosensitization, Basiliximab

## Abstract

Lymphocyte depleting induction is recommended for kidney transplant recipients (KTRs) at high immunological risk, which traditionally includes those with detectable anti-human leucocyte antigen antibodies. Data to support this approach in the modern era of histocompatibility testing are limited. We investigated outcomes in KTRs who underwent Basiliximab induction between 2012–2023 in the UK. We stratified outcomes by levels of sensitisation and T cell epitope mismatch (PIRCHE-II) scores. 1348 KTRs were included; 859 (63.7%) were unsensitised, 351 (26.0%) sensitised (calculated reaction frequency [cRF] 1%–84%), and 138 (10.3%) highly sensitised (cRF 85%–100%). Patient survival, allograft survival, and death-censored graft survival (DCGS) were 97%, 94%, and 97% at 1 year, and 88%, 78%, and 84% at 5 years respectively. There were no differences in outcomes between unsensitised and sensitised recipients; graft survival was lower in highly sensitised patients. T cell epitope mismatch scores were higher in those with rejection at 1 year (ln[PIRCHE+1] 3.94 ± 1.01 no rejection vs. 4.25 ± 0.58 rejection, p = 0.02) and epitope mismatch was associated with early rejection in multivariable analyses (Odds Ratio 1.58, 95% CI 1.01–2.62). Hence, non-depleting induction provides good outcomes in unsensitised and sensitised KTRs. T cell epitope mismatches inform rejection risk in the first post-transplant year.

## Graphical Abstract

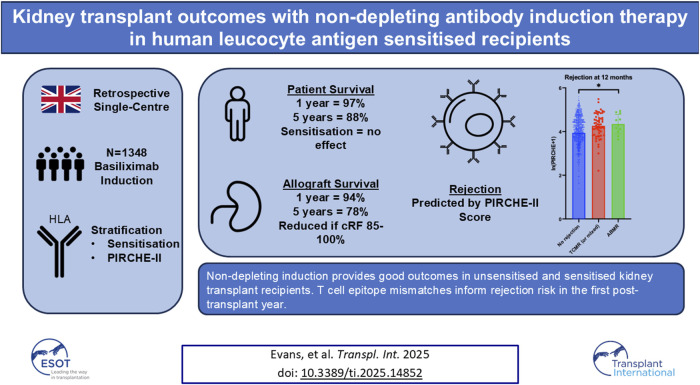

## Introduction

Induction immunosuppression in the form of antibody therapy is utilised in most kidney transplant procedures. These agents primarily modulate the T cell response to foreign human leucocyte antigens (HLAs). This results in reduced rates of acute rejection and allows for a reduction in the use of other immunosuppressive agents, such as calcineurin inhibitors and corticosteroids, that have unwanted side effects when used at high dose [1].

Induction antibody therapy may be classified into agents that deplete T cells (e.g., Antithymocyte globulin [ATG] and Alemtuzumab [Campath]), B cells or complement, and agents that are non-depleting, which act by inhibiting cytokine signalling important in T cell activation and proliferation, e.g., IL-2 receptor antagonists (IL2-RAs) such as Basiliximab. In general, depleting agents provide more profound immunosuppression which is counterbalanced by increased infectious and malignant complications as well as an increased cost [2].

The choice of which induction agent to use continues to be a source of debate amongst transplant professionals globally, with marked variation in practice between centres even within the same country [3–5]. International guidelines published by Kidney Disease Improving Global Outcomes (KDIGO) recommend basing the choice of induction agent on an assessment of immunological risk, with IL2-RAs recommended as first line, and depleting antibodies used in cases at increased risk [6]. This approach is supported by guidelines from the United Kingdom (UK) [7].

One of the key determinants of immunological risk is the presence of preformed antibodies to HLAs. Traditionally, the presence of such antibodies was detected and identified using a panel of lymphocytes, with the relatively non-specific and subjective complement dependent cytotoxicity test, which was then reported as percentage panel reactive antibodies (PRA) [8]. Significant advances in histocompatibility methods, including the development of single antigen bead testing using Luminex based technology, have meant the identification of HLA antibodies now occurs with exquisite sensitivity and specificity [9]. The presence of antibodies is now quantified by the calculated PRA (cPRA), or the calculated reaction frequency (cRF) in the UK, with immunological risk primarily due to antibodies that are donor specific [10, 11]. These advancements in HLA antibody identification methodology have occurred in parallel with advancements in molecular HLA typing methods which have enabled the HLA typing of transplant pairs at all loci to a high resolution. Subsequent computational algorithms have been developed to inform HLA matching according to differences in structure at the epitope level [12, 13].

The KDIGO guidelines, published 13 years ago, are primarily based on studies that pre-date these advancements in histocompatibility and immunogenetics [6]. For example, there have been 2 large trials comparing ATG with IL2-RA induction in patients at increased immunological risk, and inclusion was based on the historic assessment of HLA sensitisation with PRA in both [14, 15]. Moreover, the pivotal study by Brennan et al. compared ATG to Basiliximab in the setting of maintenance immunosuppression with cyclosporin, subsequently shown to be inferior to tacrolimus based regimens [16]. As such, the relevance of these historic guidelines to the contemporary management of kidney transplant recipients should be questioned, and cohorts reporting outcomes in patients managed in the modern era of histocompatibility testing are required.

For over a decade, our centre protocol has been to use IL2-RA induction for all kidney transplant recipients. This provides a unique opportunity for the assessment of kidney transplant outcomes when non-depleting induction therapy is used across a range of immunological risk. In this study, we determine patient and allograft outcomes of kidney transplants undertaken with Basiliximab induction. We assess outcomes stratified by current standard and novel measures of immune risk.

## Patients and Methods

### Study Design, Setting, and Participants

We undertook a single-centre, observational, cohort study of kidney transplant recipients who underwent transplantation at the Royal Free Hospital, London, UK. Adult patients (aged >18 years) who underwent kidney alone transplantation and had induction therapy with Basiliximab between 1st January 2012 and 31st December 2022 were included. Patients who underwent multiorgan transplant, and those who had induction with depleting antibodies, or in whom the induction agent was unclear, were excluded.

Our centre provides kidney transplant services to a multi-ethnic population from a large geographical area in north central London and Hertfordshire. Approximately 130 kidney alone transplants are undertaken each year. For the entire study period, the unit protocol was for all patients to undergo induction with Basiliximab, 20 mg administered intravenously on the day of transplant, repeated on postoperative day 4. The maintenance immunosuppression and infectious prophylaxis protocols are outlined in Supplementary File 1 [1]. Ultimately around 70% of recipients are managed steroid free long term [17]. We follow a pre-emptive strategy for the management of cytomegalovirus (CMV) and protocol biopsies are not performed. HLA antibodies are routinely measured at 1-, 3-, 6- and 12-month after transplant, and yearly thereafter. Biopsies are performed if an HLA antibody is donor specific and its development is associated with evidence of graft dysfunction (e.g., change in creatinine or development of proteinuria).

### Variables, Data Sources and Measurement

Data were documented prospectively within electronic health records and retrospectively analysed. Clinical variables related to the donor (age, sex, and donor type), recipient (age, sex, ethnicity, and cause of end stage kidney disease), and the transplant (pre-emptive, previous transplant, and mismatch at HLA-A, -B, and -DR loci) were recorded.

#### Assessment of HLA Sensitisation

Patients were grouped according to levels of HLA sensitisation. Levels of sensitisation were determined using the cRF at the time of transplantation. This measure represents the percentage of the previous 10,000 blood group identical kidney donors against whom the recipient has HLA antibodies. The inclusion of blood group distinguishes the cRF from the assessment of sensitisation using the calculated panel reactive antibody, which is the predominant method outside of the UK [18]. Details of the techniques used for antibody identification and HLA typing are outlined in Supplementary File 1 [2]. Patients were categorised as unsensitised (cRF 0%), sensitised (cRF 1%–84%), and highly sensitised (cRF 85%–100%); a subgroup analysis was undertaken in patients who were very highly sensitised (cRF 98%–100%).

#### Assessment of T cell Epitope Mismatch

In a subset of patients with the necessary molecular HLA typing, T cell epitope mismatches were determined. T cell epitope mismatches were quantified using Predicted Indirectly ReCognizable HLA Epitope (PIRCHE-II) scores.^1^ This scoring system employs a computational algorithm using *in silico* antigen presentation pathway analysis to predict the number of mismatched HLA peptides that can be presented in the context of recipient HLA class II [13]. The PIRCHE-II score is the sum of all donor-derived candidate peptides that have a predicted binding affinity to the recipients HLA class II of less than 1000 nM [19]. Scores were transformed using the natural logarithm for analysis [20], with a score of 1 added to all recipients to allow inclusion of patients with a PIRCHE-II score of 0, as has been undertaken in previous analyses [21].

### Outcome Measures

Patient and allograft outcomes were recorded at 12-, 36-, and 60-month after transplantation. Primary outcome measures were patient survival, graft survival, and death-censored graft survival. We also determined graft function (creatinine and estimated glomerular filtration rate [eGFR]), rates and type of biopsy proven rejection, rejection free allograft survival, infectious complications including CMV and BK viremia, and the development of malignancy. Outcomes were stratified by cRF and PIRCHE-II scores.

### Statistical Methods

Data are reported as number and percentages for categorical variables and mean and standard deviation (SD) or median and interquartile range (IQR) for numerical variables depending on data distribution. Categorical variables were compared using the Fisher’s exact or Chi-squared test. Numerical variables were compared between 2 groups using the Mann–Whitney or an unpaired *t* test, and across greater than 2 groups with a one-way analysis of variance. Kaplan-Meier survival curves were plotted for patient and allograft outcomes, with differences between groups assessed using the log-rank test. Multivariable logistic regression analyses were undertaken to determine clinical variables associated with rejection at 12-month. Odds ratios (OR) and 95% confidence intervals (CIs) were determined for each variable. Multivariable cox regression analyses were undertaken to determine clinical variables associated with patient survival, graft survival, DCGS, and rejection-free allograft survival over 60-month of follow-up. Hazard ratios (HR) and 95% CIs were determined for each variable. Variables included in multivariable models were recipient demographic variables and clinical variables with a p value of <0.05 in univariable analyses. These included recipient age, sex, and ethnicity, transplant type (live/DBD/DCD; pre-emptive or not; first or subsequent graft), HLA-mismatch, cRF, and ln(PIRCHE+1). Models were developed with cRF and ln(PIRCHE+1) as both continuous and categorical variables. Analysis was performed using GraphPad Prism version 10.^2^ A *p*-value of ≤0.05 was considered statistically significant.

### Ethics Statement

The study involved the retrospective analysis of routinely collected clinical data and, as such, was exempt from formal review board approval.

## Results

### Cohort Description

**FIGURE 1:**
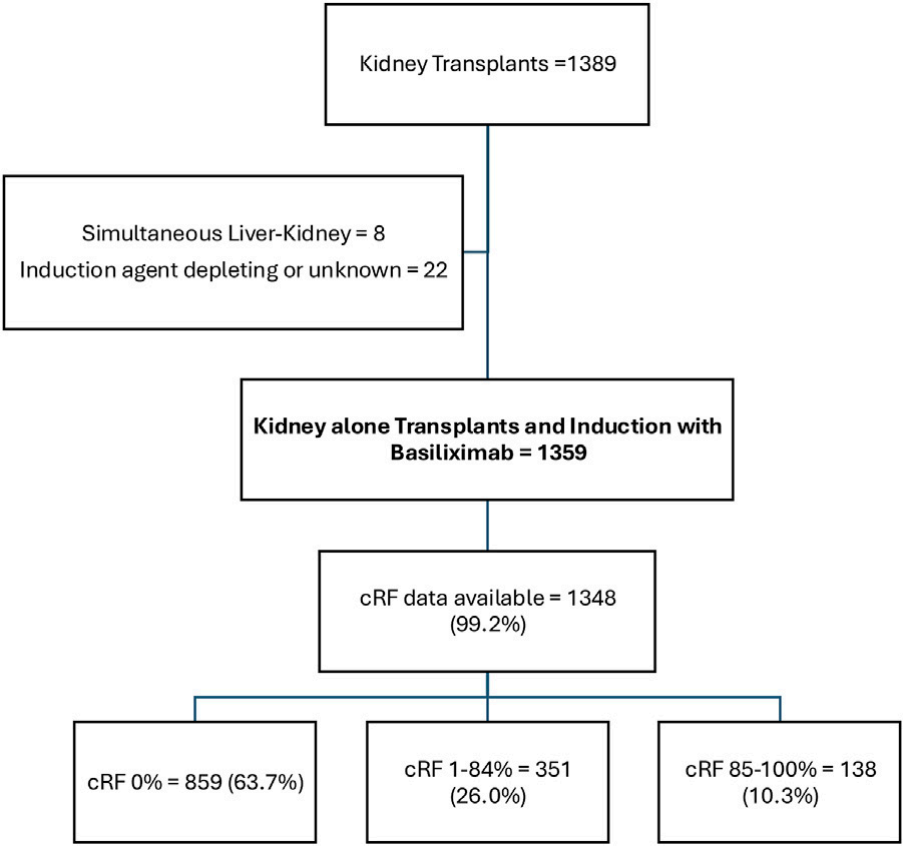
Cohort Description.

1389 kidney transplants were undertaken during the study period. Of these, 1359 (97.8%) were kidney alone transplants that underwent induction with Basiliximab and were included in the analysis (Figure 1). Recipients had a mean age of 50.0 ± 14.0 years, 494 (36.7%) were female, 615 (45.3%) were of white ethnicity, and diabetes was the cause of ESKD in 307 (22.6%) patients (Table 1). 374 (27.5%) patients underwent living donor kidney transplant, and 985 (72.5%) patients underwent deceased donor kidney transplant. Transplants were pre-emptive in 323 (23.8%) patients and represented a first kidney transplant in 1168 (86.0%) cases.

**TABLE 1:** Clinical characteristics of the cohort.

Clinical Variable	Whole population	cRF 0%	cRF 1%–84%	cRF 85%–100%	P-value
Number of patients	1359	859	351	138	
Donor Variables					
Age (mean; SD)	48.30 (14.67)	48.86 (14.60)	47.77 (14.31)	45.99 (15.81)	0.07
Donor type					
Live	374 (27.5)	254 (29.6)	102 (29.1)	18 (13.1)	**<0.0001**
Donor after Brain Death (DBD)	628 (46.2)	367 (42.7)	167 (47.6)	86 (62.3)	
Donor after Cardiac Death (DCD)	357 (26.3)	238 (27.7)	82 (23.4)	34 (24.6)	
Recipient Variables					
Age (mean; SD)	49.95 (13.98)	50.51 (14.39)	49.43 (13.34)	47.28 (13.00)	**0.032**
Sex (n = female; %)	494 (36.4)	249 (29.0)	166 (47.3)	73 (52.9)	**<0.0001**
Ethnicity					
White (n; %)	615 (45.3)	414 (48.2)	146 (41.6)	51 (37.0)	**0.0063**
Asian (n; %)	408 (30.0)	255 (29.7)	113 (32.2)	38 (27.5)	
Black (n; %)	336 (24.7)	190 (22.1)	92 (26.2)	49 (35.5)	
Cause of ESKD					
Diabetes (n; %)	307 (22.6)	217 (25.3)	64 (18.2)	23 (18.2)	**0.0094**
Polycystic kidney (n; %)	105 (7.7)	73 (8.5)	22 (8.5)	10 (6.3)	
Other/unknown (n: %)	947 (69.7)	569 (66.2)	265 (66.2)	105 (75.5)	
Transplant Variables					
Pre-emptive (n; %)	323 (23.8)	219 (25.5)	85 (22.9)	16 (11.5)	**0.0008**
First transplant (n; %)	1168 (86.0)	821 (95.6)	288 (82.1)	51 (37.0)	**<0.0001**
Total HLA-A, -B, -DR Mismatch (mean; SD)	2.99 (1.37)	3.08 (1.31)	2.96 (1.37)	2.41 (1.49)	**<0.0001**
Total HLA-A, -B, -DR Mismatch 0–3 (n; %)	900 (66.2)	561 (65.3)	231 (65.8)	105 (76.1)	**0.0396**

Significant results are highlighted in bold.

cRF data were available in 1348 (99.2%) patients. 859 (63.7%), 351 (26.0%), and 138 (10.3%) patients had cRFs of 0% (unsensitised), 1%–84% (sensitised), and 85%–100% (highly sensitised) respectively. 59 (4.4%) patients had a donor specific antibody (DSA) detectable at the time of transplant (antibodies against all HLA loci were represented, median fluorescence intensity ranged 971–8000), and 36 (2.8%) patients had a DSA detectable in historical sera. Highly sensitised patients were younger, more commonly female, less commonly white, less frequently underwent living or pre-emptive kidney transplantation, and had a better total match at HLA-A, -B, and -DR loci.

### Patient and Allograft Outcomes

**FIGURE 2:**
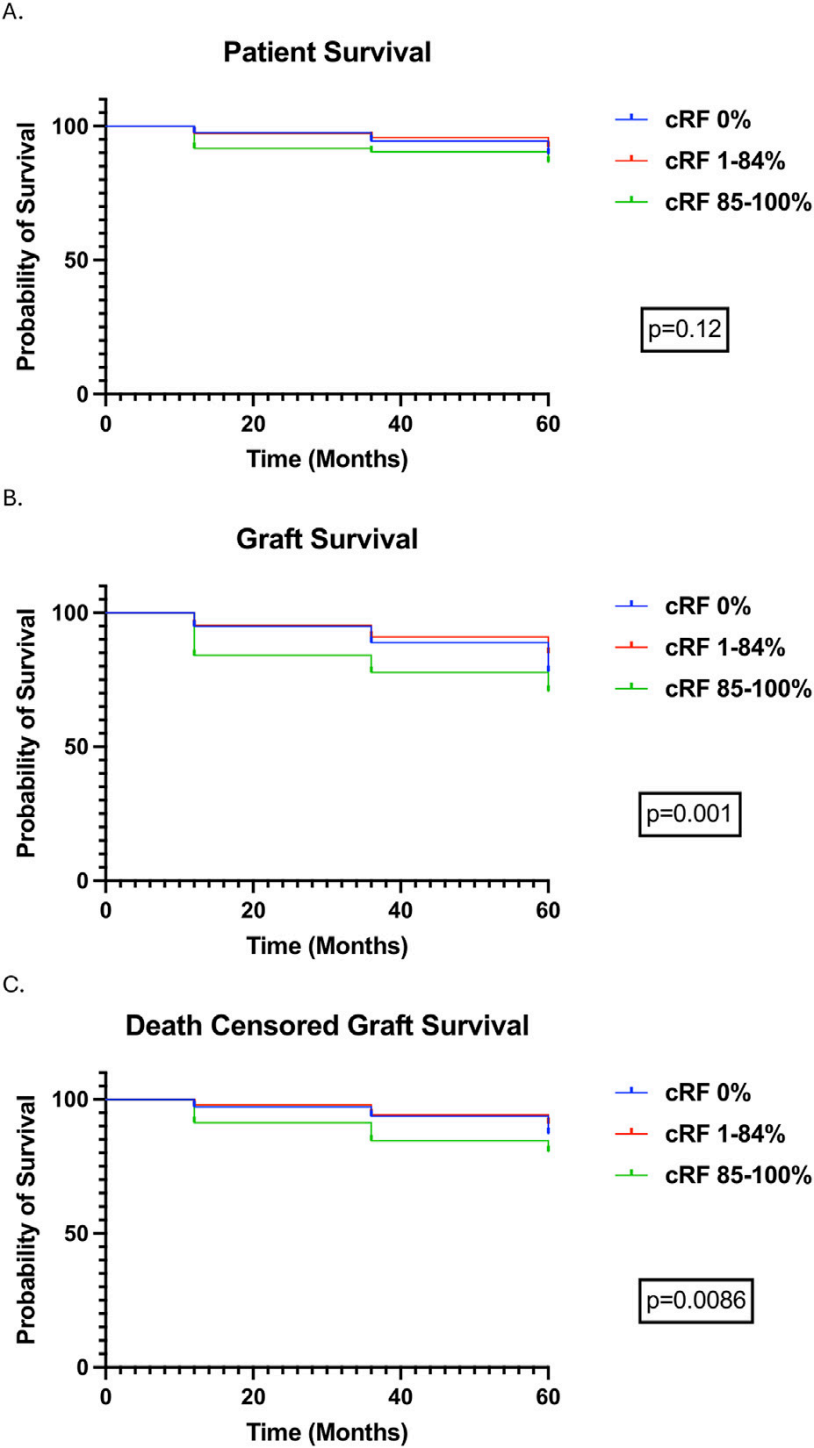
Transplant Outcomes over a follow-up period of 5 years stratified by cRF. Survival curves were compared using the logrank test. **(A)** Patient Survival, **(B)** Allograft Survival, **(C)** Death Censored Allograft Survival.

1080 (79.5%) patients were followed up to 12 months, 844 (62.1%) patients to 36 months, and 659 (48.5%) patients to 60 months. In the entire cohort, patient survival was 96.9%, 93.6%, and 87.6%, allograft survival was 93.9%, 88.4%, and 78.3%, and DCGS was 96.9%, 92.1%, and 84.5% at 12-, 36-, and 60-month respectively (Table 2). Patient survival was not different according to cRF categories, whereas allograft survival and DCGS were lower in highly sensitised patients compared to other cRF groups (Figure 2). Subgroup analyses of outcomes in very highly sensitised patients (cRF 98%–100%) and in deceased donor kidney transplants are outlined in Supplementary File 1 [3], [4]; patient and allograft outcomes followed similar trends to the wider cohort, albeit did not reach statistical significance for all outcomes. There was no difference in patient or allograft survival between sensitised patients with and without a preformed DSA (detected either at the time of transplant or historically).

**TABLE 2:** Patient and Allograft outcomes at 12-, 36-, and 60-month in the whole population, and in unsensitised (cRF 0%), sensitised (cRF 1%–84%), and highly sensitised (cRF 85%–100%) patients.

Outcome	Whole population	cRF 0%	cRF 1%–84%	cRF 85%–100%	P-value (comparing all cRF categories)	P-value (cRF 0% vs. cRF 1%–84%)	P-value (cRF 0% vs. cRF 85%–100%)	P-value (cRF 1%–84% vs. cRF 85%–100%)
Patient survival								
12 months	1047/1080 (96.94)	653/669 (97.61)	290/298 (97.32)	104/113 (92.04)	0.16	0.82	**0.0054**	**0.02**
36 months	790/844 (93.60)	482/514 (93.77)	240/252 (95.24)	68/78 (87.18)	0.39	0.51	0.054	**0.019**
60 months	577/659 (87.56)	349/400 (87.25)	183/202 (90.59)	45/57 (78.95)	0.34	0.28	0.10	**0.02**
Graft Survival								
12 months	1014/1080 (93.89)	635/669 (94.92)	284/298 (95.30)	95/113 (84.07)	**0.0018**	0.87	**0.001**	**0.0004**
36 months	746/844 (88.39)	455/514 (88.52)	229/252 (90.87)	62/78 (79.49)	0.16	0.38	**0.04**	0.0092
60 months	516/659 (78.30)	306/400 (76.50)	170/202 (84.15)	40/57 (70.18)	**0.027**	**0.034**	0.32	**0.02**
Death Censored Allograft Survival								
12 months	1014/1047 (96.85)	635/653 (97.24)	284/290 (97.93)	95/104 (91.35)	0.09	0.66	**0.007**	**0.0053**
36 months	746/810 (92.10)	455/490 (92.86)	229/244 (93.86)	62/76 (81.58)	**0.0324**	0.76	**0.0033**	**0.0024**
60 months	516/611 (84.45)	306/364 (84.07)	170/191 (89.01)	40/56 (71.43)	**0.0079**	0.13	**0.04**	**0.0024**

Significant results are highlighted in bold.

### Graft Function, Rejection, Infection and Malignancy

Median (IQR) creatinine of all patients was 125 (102–158) μmol/L, 129 (102–175) μmol/L, and 130 (102–187) μmol/L at 12-, 36-, and 60-month post-transplant; eGFR was 50 (38–64) mL/min, 47 (33–64) mL/min, and 48 (32–64) mL/min at the same timepoints respectively (Table 3; Supplementary File 1 [5]). There were no differences in GFR between cRF categories at any of the follow-up time points (Table 3, Supplementary File 1 [6]).

**TABLE 3:** Outcomes 12-, 36-, and 60-month in unsensitised (cRF 0%), sensitised (cRF 1%–84%), and highly sensitised (cRF 85%–100%) recipients.

Outcomes	Whole population	cRF 0%	cRF 1%–84%	cRF 85%–100%	P-value (comparing all cRF categories)	P-value (cRF 0% vs. cRF 1%–84%)	P-value (cRF 0% vs. cRF 85%–100%)	P-value (cRF 1%–84% vs. cRF 85%–100%)
12-month outcomes								
Creatinine (μmol/l; median, IQR)	125 (102–158)	129 (104–162)	118 (98–145)	126 (96–166)	**0.0009**	**0.0005**	>0.99	0.26
eGFR (ml/min; median, IQR)	50 (38–65)	50 (38–64)	52 (40–66)	47 (36–69)	0.19	0.29	>0.99	0.51
Rejection (n; %)	101/1080 (9.35)	63/669 (9.42)	20/298 (6.71)	18/113 (15.93)	**0.021**	0.17	**0.044**	**0.0068**
TCMR (n; % of rejection)	81/101 (80.20)	56/63 (88.89)	13/20 (65.00)	12/18 (66.67)	**0.014**	**0.03**	**0.03**	>0.99
ABMR (n; % of rejection)	9/101 (8.91)	2/63 (3.17)	4/20 (20.00)	3/18 (16.67)	**0.020**	**0.03**	**0.07**	>0.99
Mixed/Both TCMR and ABMR (n; % of rejection)	11/101 (10.89)	5/63 (7.94)	3/20 (15.00)	3/18 (16.67)	0.42	0.39	0.37	>0.99
CMV viremia (n; %)	259/1080 (23.98)	154/669 (23.02)	77/298 (25.84)	27/113 (23.89)	0.63	0.37	0.81	0.80
BK viremia (any level) (n; %)	137/1080 (12.69)	90/669 (13.45)	30/298 (10.07)	17/113 (15.04)	0.24	0.17	0.66	0.17
BK viremia (>10,000 copies/mL) (n; %)	65/1080 (6.02)	44/669 (6.58)	13/298 (4.36)	8/113 (7.08)	0.35	0.24	0.84	0.31
36-month outcomes								
Creatinine (μmol/l; median, IQR)	129 (102–175)	132 (107–179)	121 (96–165)	145 (102–193)	**0.0039**	**0.006**	>0.99	0.07
eGFR (ml/min; median, IQR)	47 (33–64)	47 (32–62)	50 (35–68)	41 (28–69)	0.089	0.21	>0.99	0.19
Rejection (n; %)	87/844 (10.31)	50/514 (9.73)	17/252 (6.75)	20/78 (25.64)	**<0.0001**	0.22	**0.0002**	**<0.0001**
TCMR (n; % of rejection)	66/87 (75.86)	43/50 (86.00)	9/17 (52.94)	14/20 (70.00)	**0.020**	**0.015**	0.17	0.32
ABMR (n; % of rejection)	7/87 (8.05)	1/50 (2.00)	3/17 (17.65)	3/20 (15.00)	**0.026**	**0.048**	0.07	>0.99
Mixed/Both TCMR and ABMR (n; % of rejection)	14/87 (16.09)	6/50 (12.00)	5/17 (29.41)	3/20 (15.00)	0.22	0.13	0.71	0.43
Malignancy (n; %)	52/844 (6.16)	37/514 (7.20)	10/252 (3.97)	5/78 (6.41)	0.21	0.11	>0.99	0.36
Cardiovascular event (n; %)	34/844 (4.03)	19/514 (3.70)	12/252 (4.76)	3/78 (3.85)	0.74	0.56	>0.99	>0.99
60-month outcomes								
Creatinine (μmol/l; median, IQR)	130 (102–187)	132 (105–192)	128 (98–171)	122 (96–201)	0.15	0.19	>0.99	>0.99
eGFR (ml/min; median, IQR)	48 (32–64)	47 (31–63)	50 (32–66)	46 (28–72)	0.64	>0.99	>0.99	>0.99
Rejection (n; %)	76/659 (11.53)	45/400 (11.25)	16/202 (7.92)	15/57 (26.32)	**0.0016**	0.25	**0.005**	**0.0007**
TCMR (n; % of rejection)	55/76 (72.37)	33/45 (73.33)	10/16 (62.50)	12/15 (80.00)	0.56	0.53	0.74	0.43
ABMR (n; % of rejection)	8/76 (10.53)	5/45 (11.11)	1/16 (6.25)	2/15 (13.33)	0.88	>0.99	>0.99	0.60
Mixed/Both TCMR and ABMR (n; % of rejection)	13/76 (17.11)	7/45 (15.56)	5/16 (31.25)	1/15 (6.67)	0.22	0.27	0.67	0.17
Malignancy (n; %)	53/659 (8.04)	34/400 (8.50)	13/202 (6.44)	6/57 (10.53)	0.51	0.42	0.62	0.39
Cardiovascular event (n; %)	39/659 (5.91)	23/400 (5.75)	14/202 (6.93)	2/57 (3.51)	0.67	0.59	0.76	0.54

Significant results are highlighted in bold.

**FIGURE 3:**
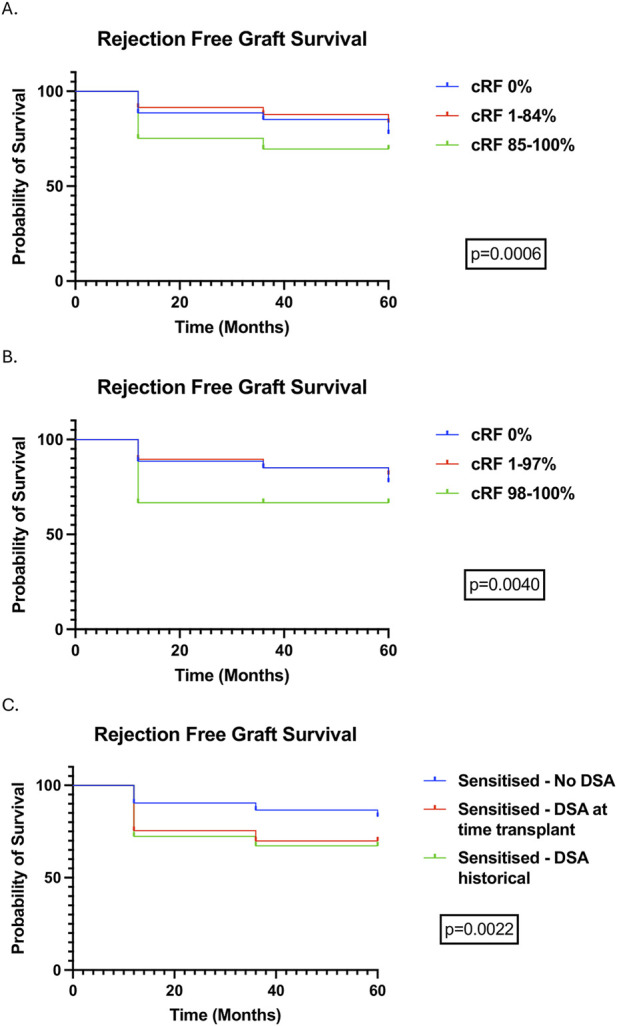
Rejection free allograft survival (censored for death) stratified by cRF categories, demonstrating outcomes in highly sensitised **(A)** and very highly sensitised **(B)** patients, and stratified by the presence of DSAs, detectable at the time of transplant and historically **(C)**.

Rejection rates (cumulative) were 9.35%, 10.31%, and 11.53% in patients followed-up to 12-, 36-, and 60-month. Rejection was more common in highly sensitised patients at all time points and there was more antibody mediated rejection (ABMR) within the first 12 months as levels of sensitisation increased (Table 3). 11 (61%) of 18 highly sensitised patients that experienced rejection at 12-month were very highly sensitised (cRF 98%–100%); outcomes in very highly sensitised patients are summarised in Supplementary File 1 [7]. As with highly sensitised patients, rejection was higher in very highly sensitised compared to other cRF categories at all time points, and ABMR occurred more frequently, albeit T cell mediated rejection (TCMR) remained the commonest type of rejection overall. Rejection free allograft survival was worse in highly sensitised and very highly sensitised patients compared to other cRF groups, primarily driven by increased rejection in the first post-transplant year (Figures 3A,B). Rejection free allograft survival was also worse in sensitised patients with a preformed DSA detected either at the time of transplant or in historical sera, compared to sensitised patients without a preformed DSA (Figure 3C).

CMV viremia occurred in 259 (23.98%) patients within the first post-transplant year. BK viremia of any level occurred in 137 (12.69%) patients and BK viremia >10^4^ copies/mL occurred in 65 (6.02%) patients. There was no difference in the prevalence of either infection between cRF categories (Table 3). At 60-month post-transplant, 53 (8.04%) patients had developed a malignancy, and 39 (5.91%) patients had experienced a cardiovascular event. There were also no differences in these events between cRF categories (Table 3).

### T cell Epitope Mismatch and Rejection

**FIGURE 4:**
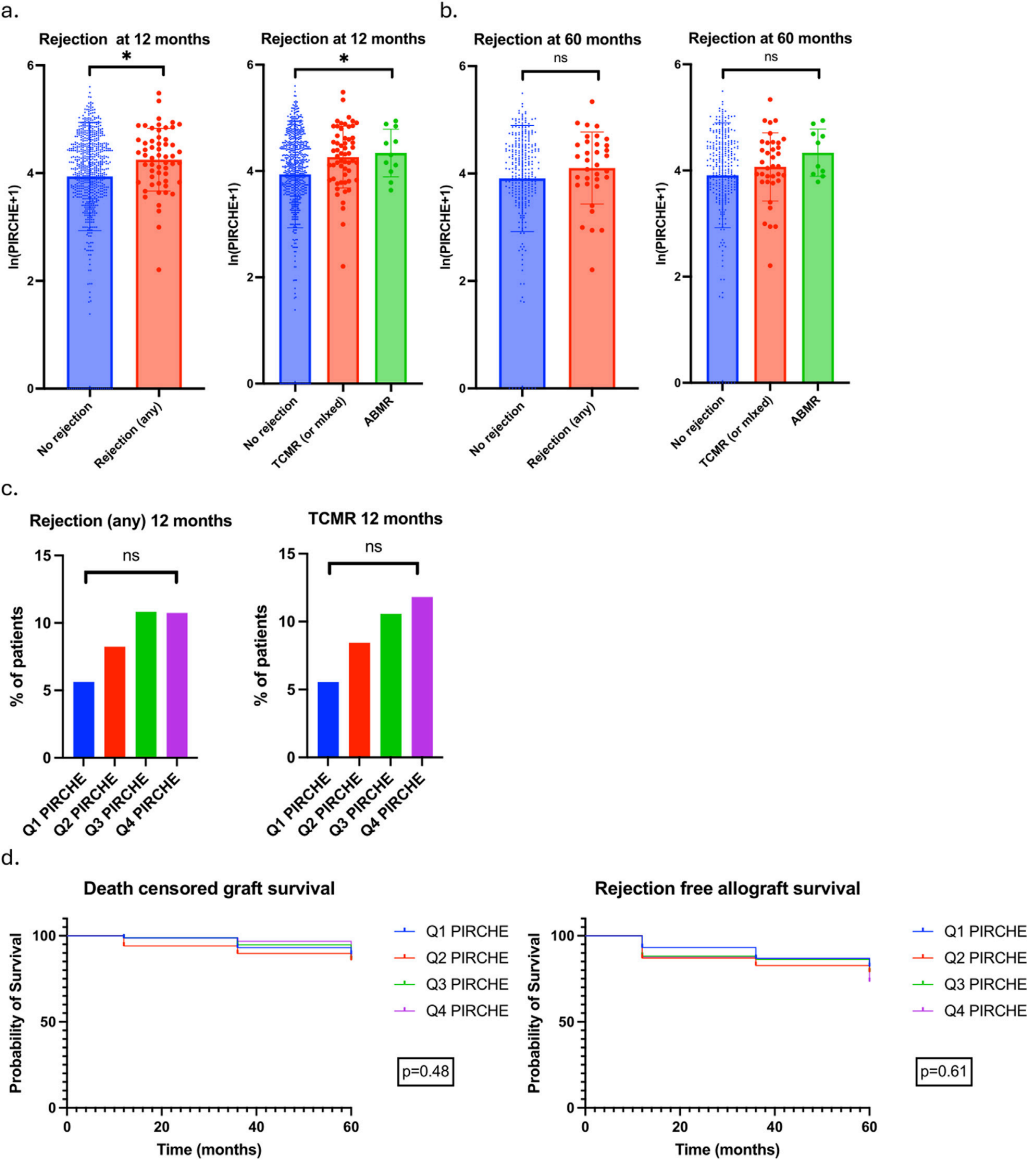
Association between PIRCHE scores and rejection. **(a)** ln(PIRCHE+1) scores in patients with no rejection, any rejection, TCMR/mixed rejection and ABMR at 12 months. Mean and SD values plotted. No rejection 3.937 ± 1.005, Any rejection 4.249 ± 0.583, TCMR/mixed 4.262 ± 0.581, ABMR 4.330 ± 0.447. P value for unpaired T-test comparing No rejection to any rejection 0.022; P value for one-way ANOVA comparing no rejection and TCMR/mixed and ABMR 0.024. **(b)** ln(PIRCHE+1) scores in patients with no rejection, any rejection, TCMR/mixed rejection and ABMR at 60 months. Mean and SD values plotted. No rejection 3.909 ± 0.987, Any rejection 4.102 ± 0.669, TCMR/mixed 4.067 ± 0.644, ABMR 4.336 ± 0.444. P value for unpaired T-test comparing No rejection to any rejection 0.27; P value for one-way ANOVA comparing no rejection and TCMR/mixed and ABMR 0.27. **(c)** 12-month rejection rates in patients divided into PIRCHE score quartiles. Rates of any rejection and TCMR/mixed rejection plotted. **(d)** DCGS and rejection free allograft survival in patients stratified by PIRCHE quartile.

T cell epitope mismatch data were available in 825 patients; the baseline clinical characteristics of these patients are outlined in Supplementary File 1 [8]. 740 (89.7%) and 335 (40.6%) patients completed follow-up to 12 and 60 months respectively. Mean ln(PIRCHE+1) scores were 4.249 ± 0.583 and 3.973 ± 1.005 in patients with and without rejection at 12-month (p = 0.022), and 4.102 ± 0.669 and 3.909 ± 0.987 in patients with and without rejection at 60-month (p = 0.27) (Figures 4a, b).

Patients were divided into PIRCHE score quartiles with quartile 1 (Q1 PIRCHE) having the lowest and quartile 4 (Q4 PIRCHE) having the highest PIRCHE scores. Rejection of any type at 12 months occurred in 9 (5.63%) patients in Q1 PIRCHE and 16 (10.74%) patients in Q4 PIRCHE (p = 0.29); TCMR occurred in 8 (5.56%) and 15 (11.81%) patients in Q1 and Q4 PIRCHE respectively (p = 0.27). There was a stepwise increase in rejection (both any rejection and TCMR) with each increase in PIRCHE quartile but this did not reach statistical significance (Figure 4c). There were no differences in DCGS or rejection free allograft survival when patients were stratified by PIRCHE scores (Figure 4d).

Multivariable logistic regression analyses were undertaken to determine clinical variables associated with rejection at 12-month (Table 4). Ln(PIRCHE+1) was associated with rejection at 12-month (Odds Ratio 1.576, 95% CI 1.006–2.618) whereas cRF was not.

**TABLE 4:** Multivariable logistic regression analyses of clinical variables associated with rejection (any type) at 12-month. Odds ratios and 95% confidence intervals are provided for each variable included within the model. cRF is included as a categorical variable and ln(PIRCHE+1) as a continuous variable.

Clinical Variable	Odds Ratio for Rejection at 12-month	95% confidence interval
Age at Transplant	0.9844	0.9629 to 1.006
Male Sex [reference = female]	0.7775	0.4319 to 1.423
Ethnicity [black; reference = white]	0.9112	0.4449 to 1.825
Ethnicity [Asian; reference = white]	0.7587	0.3572 to 1.548
DBD transplant [reference = live transplant]	0.5766	0.2690 to 1.246
DCD transplant [reference = live transplant]	0.9076	0.4140 to 2.005
Total HLA Mismatch at HLA-A, -B, -DR loci	1.060	0.8121 to 1.382
cRF 1%–84% (sensitised) [reference = unsensitised)	0.6298	0.2819 to 1.296
cRF 85%–100% (highly sensitised) [reference = unsensitised)	1.856	0.7282 to 4.475
Pre-emptive transplant [reference = not pre-emptive]	1.336	0.6619 to 2.586
Multiple grafts [reference = first graft]	1.271	0.5613 to 2.708
ln(PIRCHE+1)	**1.576**	**1.006 to 2.618**

Significant results are highlighted in bold.

### Multivariable Analyses of Patient and Graft Outcomes

Cox regression analyses were undertaken to determine clinical variables associated with patient survival, graft survival, DCGS, and rejection-free allograft survival (Table 5; Supplementary File 1 [9], [10]). A higher PIRCHE score (HR 1.350, 95% CI 1.028–1.817) was associated with worse rejection-free allograft survival, whereas cRF was not associated with any outcome.

**TABLE 5:** Cox regression analyses of clinical variables associated with patient survival, graft survival, death-censored graft survival and rejection-free allograft survival. Hazard ratios and 95% confidence intervals are provided for each variable included within the model. cRF is included as a categorical variable and ln(PIRCHE+1) as a continuous variable.

Clinical Variable	Patient survival		Graft survival		Death-censored graft survival		Rejection-free allograft survival	
	Hazard ratio	95% confidence interval	Hazard ratio	95% confidence interval	Hazard ratio	95% confidence interval	Hazard ratio	95% confidence interval
Age at Transplant	**1.069**	**1.040 to 1.102**	**1.025**	**1.008 to 1.042**	1.002	0.9805 to 1.025	0.9884	0.9735 to 1.003
Male Sex [reference = female]	1.168	0.6102 to 2.357	0.7787	0.5059 to 1.211	**0.5220**	**0.2934 to 0.9274**	**0.6102**	**0.4110 to 0.9089**
Ethnicity [black; reference = white]	**0.2849**	**0.09454 to 0.7038**	0.7934	0.4692 to 1.318	1.392	0.7266 to 2.690	1.051	0.6621 to 1.658
Ethnicity [Asian; reference = white]	0.8578	0.4248 to 1.673	0.7817	0.4656 to 1.287	0.7682	0.3573 to 1.590	0.7088	0.4213 to 1.164
DBD transplant [reference = live transplant]	1.456	0.5695 to 4.503	1.639	0.8542 to 3.423	1.837	0.7645 to 5.155	0.9129	0.5388 to 1.579
DCD transplant [reference = live transplant]	0.9462	0.3476 to 3.023	1.772	0.8911 to 3.802	**2.906**	**1.179 to 8.311**	1.340	0.7796 to 2.346
Total HLA Mismatch at HLA A-, B-, and DR-loci	0.9653	0.7345 to 1.266	0.8341	0.6869 to 1.011	0.7680	0.5869 to 1.002	0.9243	0.7672 to 1.111
cRF 1%–84% [reference = cRF 0%)	0.9219	0.4399 to 1.828	0.8069	0.4804 to 1.315	0.7633	0.3803 to 1.461	0.6852	0.4154 to 1.097
cRF 85%–100% [reference = cRF 0%)	0.6697	0.1315 to 2.542	1.138	0.4991 to 2.407	1.348	0.5105 to 3.231	1.600	0.8361 to 2.918
Pre-emptive transplant [reference = not pre-emptive]	**0.07196**	**0.004035 to 0.3371**	**0.3434**	**0.1504 to 0.6822**	0.5652	0.2242 to 1.228	1.123	0.6878 to 1.779
Multiple grafts [reference = first graft]	1.747	0.5514 to 4.799	1.093	0.5253 to 2.128	0.9386	0.3539 to 2.181	1.277	0.7167 to 2.181
ln(PIRCHE+1) as continuous variable	1.197	0.7712 to 1.942	1.140	0.8571 to 1.551	1.124	0.7819 to 1.679	**1.350**	**1.028 to 1.817**

Significant results are highlighted in bold.

## Discussion

### Key Results

Induction immunosuppression is widely used in kidney transplantation but there is marked variation in which induction regimen is used. The relevance of guidelines that recommend depleting antibody induction based on traditional assessments of immune risk to the contemporary management of kidney transplant recipients is unknown. We therefore assessed outcomes in kidney transplant recipients who underwent induction with non-depleting antibody therapy in the modern era of histocompatibility testing, with outcomes stratified by HLA sensitisation determined by single antigen bead testing, and T cell epitope mismatches determined by PIRCHE-II scores.

We included an ethnically diverse cohort of 1359 kidney transplant recipients who underwent induction with Basiliximab. Just over one third of the cohort were sensitised, and 1 in 10 recipients were highly sensitised to HLA antigens. Patient survival, graft survival and DCGS were 88%, 78%, and 84% at 5 years respectively, representing favourable outcomes compared to registry data [22, 23]. There were no differences in these primary outcomes in sensitised compared to unsensitised recipients; a reduction in graft survival and DCGS were restricted to highly sensitised recipients but remained 70% and 71% at 5 years. The 12-month rejection rate was 9% overall and the major rejection type was TCMR. As was seen with the outcome data, there was no difference in rejection rates in sensitised compared to unsensitised recipients; there was an increase in rejection restricted to the highly sensitised group at all follow-up timepoints, with a cumulative rejection rate of 26% in highly sensitised recipients who were followed up to 5 years. Rejection free allograft survival was worse in sensitised patients with a preformed DSA. The association of T cell epitope mismatches with outcomes was assessed in 825 recipients and there was an increase in PIRCHE-II scores in those with rejection in the first year. T cell epitope mismatch, but not cRF, was associated with early rejection and rejection free allograft survival in multivariable analyses.

### Interpretation

The 2012 KDIGO guidelines on the management of kidney transplant recipients recommends basing the choice of induction therapy on an assessment of immunological risk. Non-depleting antibody therapy is recommended first line, and depleting antibody induction is recommended for patients at high immunological risk, defined by several factors including any level of HLA sensitisation [6]. This recommendation is underpinned by a meta-analysis published shortly before guideline development that highlighted a 25% reduction in graft loss at 1 year with IL2-RA compared with no antibody induction, and a 30% increase in risk of biopsy-proven acute rejection at 1 year when IL2-RA was compared to ATG; this came at the cost of a 75% increase in malignancy and 32% increase in CMV disease [2]. Two multicentre randomised studies have compared IL2-RA induction with ATG specifically in patients at increased immunological risk, with one of these studies making this comparison in the setting of cyclosporin based immunosuppression [14, 15]. Both studies demonstrated a reduction in rejection with ATG compared to IL2-RA at 1- and 5-years, but importantly no difference in patient or allograft outcomes were demonstrated, with follow up now reported out to 10 years [24–26]. A comparison of IL2-RA with ATG induction coupled with early steroid withdrawal in a predominantly white low immunological risk population was made in the Harmony study, which demonstrated no difference in rejection rates, patient or allograft outcomes at 1- and 5-years between the arms [27, 28]. A more recent pilot study demonstrated no difference between depleting and non-depleting induction in sensitised recipients without preformed DSAs [29]. The lack of proven benefit of depleting antibody induction on hard outcomes (i.e., patient and allograft survival), coupled with a more adverse side effect profile and cost, underlies our unit policy for Basiliximab induction in all. In this study we provide unique real-world data on outcomes from the use of this uniform approach in a large contemporary cohort of patients undergoing kidney transplantation across a range of immunological risk.

The outcomes of this strategy are summarised in Supplementary File 2 and outlined alongside those seen in previous large randomised controlled trials of induction therapy that include high [14, 15, 24–26], low [16, 27, 28, 30, 31], and mixed [32, 33] immunological risk populations, in addition to recent registry data from the US [22] and the UK [23], and other large registry analyses [34, 35]. Patient survival in our cohort was 96.9% at 1-year and 87.6% at 5-years (96.1% and 85.4% in deceased donors), similar to patient outcomes previously reported. For example, patient survival was 95%–97% at 1-year and 85%–90% at 5-years in the low immunological risk Harmony population [27, 28]; UK-wide patient survival after deceased donor kidney transplant is currently 96% and 85% at 1- and 5-years respectively [23]. Graft survival (censored for death) was 96.9% and 84.5% at 1- and 5-years (96.0% and 82.1% in deceased donors), providing favourable outcomes compared to recently reported 5-year US graft survival of 66.1%–82.2% after deceased donor kidney transplantation [22]. Graft survival in our cohort did not differ in sensitised compared to unsensitised recipients, and in highly sensitised recipients was 91.4% and 71.4% at 1- and 5-years. These graft outcomes are similar to the outcomes in the high immunological risk population that underwent induction with ATG in the TAXI study, where graft survival was 85% and 76% at 1- and 5-years [15, 25]. Hence, the use of non-depleting antibody induction in our cohort in recipients across a range of immunological risk provided comparable patient and graft outcomes to previous cohorts where depleting antibody induction has been used. This occurred in an ethnically diverse population, which was predominantly managed steroid free.

Acute rejection rates have steadily declined over the last 2 decades. In 2000, rejection within the first year occurred in 17%–24% of kidney transplant recipients in the US [35], and early rejection occurred in 15%–16% of high immunological risk patients managed with depleting antibodies and 26%–27% of patients managed with non-depleting antibodies in the older clinical trials [14, 15]. 1-year rejection rates reduced to 8%–10% in the US in 2012, and the most recent data demonstrate rates of 5.7% and 6.0% in patients undergoing induction with non-depleting and depleting induction respectively [22]. The current low rates of acute rejection encountered in routine clinical practice may lead us to question whether this outcome remains as relevant in contemporary analyses and clinical trials. Rejection within the first year in our cohort occurred in 9.4% of patients, consistent with US registry data from the last decade, and the 11% acute rejection rate seen in Harmony when Basiliximab induction was combined with a steroid free regimen [27]. Clinically relevant BK viremia (>10^4^ copies/mL) was relatively infrequent (6%) in our cohort, and the non-depleting induction facilitated a pre-emptive approach to CMV management with acceptable rates of viremia. Cumulative 5-year rejection rates were 15% in Harmony [28], and 24.2% when IL2-RA induction was combined with steroid withdrawal in the Astellas corticoid study group trial [30, 31], with both these studies investigating populations at low immunological risk. Biopsy proven rejection within the first 5 years occurred in 11.5% of our cohort, despite the inclusion of patients across a range of HLA sensitisation. Two thirds of our cohort had a total HLA A-, B-, DR-mismatch of 3 or less, and our outcomes would support the current UK practice of including HLA matching within allocation schemes, especially in younger recipients. Our data would suggest that acceptable rejection rates and good graft outcomes are achievable with non-depleting induction in all when HLA antibody detection occurs using sensitive methods and efforts are made to HLA match transplant pairs.

Stratification of immunological risk in transplantation has traditionally involved an assessment of the total level of sensitisation to HLA antibodies [36]. Guidelines suggest that any level of sensitisation leads to increased risk and the need for depleting antibody induction, but our data would not support this given no difference in rejection rates or graft outcomes between unsensitised and sensitised recipients. Moreover, cRF was not associated with rejection or graft outcomes in multivariable models. An increase in rejection and a reduction in graft survival was seen, however, in highly sensitised recipients.

Donor specificity of antibodies is a key determinant of immunological risk and previous studies provide conflicting evidence regarding outcomes on the use of Basiliximab in the setting of pre-existing DSAs. Some reports demonstrate that in the absence of a preformed DSA, sensitisation does not impact rejection and outcomes after Basiliximab induction [29, 37], whereas others have found that it does [38]. Our data demonstrate worse rejection free allograft survival in the presence of a preformed DSA, but no difference in other patient or allograft outcomes. Whether depleting induction therapy would have improved outcomes in the highly sensitised subgroup or in patients with a preformed DSA is not answered by this study.

Given most rejection episodes were T cell mediated across all cRF categories, we investigated if T cell epitope mismatches, determined using PIRCHE scores, were associated with rejection. These mismatches predict the risk of *de novo* T cell alloimmune responses, and higher mismatch scores have previously been associated with the development of DSAs, rejection, and graft survival in kidney transplantation [20, 39, 40]. Moreover, scores have been shown to provide additive information to traditional HLA matching [41], especially in sensitised recipients, and they may inform outcomes in patients with TCMR [42, 43]. Our data support a role for T cell epitope mismatch scores in the immunological assessment of transplant pairs undergoing non-depleting antibody induction. Scores were higher in patients with rejection at 1 year and were associated with early rejection and rejection free allograft survival in multivariable models, whereas levels of sensitisation were not.

### Limitations

In this study, we provide unique data on the use of non-depleting antibody induction in a large, ethnically diverse, cohort of kidney transplant recipients with outcomes stratified by HLA sensitisation at the time of transplant and T cell epitope mismatch scores. We report these outcomes from a centre that manages most patients steroid free, albeit we lack data on the maintenance immunosuppression regimens used at the individual level. We provide medium term outcomes to 5 years but lack outcome data thereafter. We anticipate our outcomes are generalizable to many healthcare systems, albeit the ethnic diversity of the cohort, the inclusion of HLA matching within organ allocation, and the ability to perform HLA typing at medium to high resolution may mean it is not applicable to all settings. Moreover, our results should be interpreted in the knowledge that there were relatively few patients in the highly sensitised group, and a larger number of patients would be required to confirm the findings of multivariable analyses. We assess the impact of T cell epitope mismatches in a large proportion of the cohort, but not all. We report rejection that was clinically apparent, but our lack of protocol biopsies may underestimate true prevalence [44]. Moreover, we lack data on the development of DSAs, which has been shown to be impacted by choice of induction therapy, especially in sensitised recipients [45]. We provide data on some complications of immunosuppression, including infection, malignancy, and cardiovascular disease, but we lack more granular data on the development of diabetes, hyperlipidaemia and blood pressure control, which are increasingly relevant to the transplant population we manage. Moreover, we lack patient reported outcomes on the uniform use of this non-depleting induction strategy.

## Conclusion

In summary, non-depleting antibody induction provides good outcomes for kidney transplant recipients managed using contemporary histocompatibility techniques across a range of immunological risk. Depleting antibody induction may not be necessary in all patients who are sensitised to HLA antigens, but may be considered in highly sensitised recipients and those with a preformed DSA. T cell epitope mismatch scores provide useful information during the immunological assessment of transplants being undertaken with non-depleting antibody induction. We propose that guidelines for induction therapy in kidney transplantation should be reviewed and updated.

## Data Availability

The raw data supporting the conclusions of this article will be made available by the authors, without undue reservation.

## Footnotes

1
https://www.pirche.com

2
http://www.graphpad.com

## Supplementary Material

SUPPLEMENTARY FILE S2 Summary of outcomes in the cohort in this study, and in cohorts from previous randomised trials and registry analyses.

## Abbreviations

ABMR, Antibody mediated rejection; ATG, Anti-thymocyte globulin; CI, Confidence interval; CMV, Cytomegalovirus; cPRA, calculated panel reactive antibodies; cRF, calculated reaction frequency; DBD, Donor after brain death; DCD, Donor after cardiac death; DCGS, Death censored graft survival; DSA, Donor-specific antibody; eGFR, estimated glomerular filtration rate; HLA, Human leucocyte antigen; HR, Hazard ratio; IL2-RA, Interleukin-2 receptor antagonist; IQR, Interquartile range; KDIGO, Kidney Disease Improving Global Outcomes; KTR, Kidney transplant recipient; OR, Odds ratio; PIRCHE, Predicted Indirectly ReCognizable HLA Epitope; SD, Standard deviation; TCMR, T cell mediated rejection; UK, United Kingdom; US, United States.
