# Supplementary material for: Kidney Transplant Outcomes With Non-Depleting Antibody Induction Therapy in Human Leucocyte Antigen Sensitised Recipients
Source: Transpl Int. 2025 Sep 30;38:14852. doi: 10.3389/ti.2025.14852 (PMC12518175; doi:10.3389/ti.2025.14852)
Supplement: Supplementary file 1 [file Supplementaryfile1.docx]

**Supplementary 1:** Immunosuppression and infection prophylaxis protocol at the Royal Free Hospital, UK

| *Induction immunosuppression* | Basiliximab, 20mg administered intravenously on the day of transplant, repeated on postoperative day 4.  Intravenous methylprednisolone 500mg at induction, followed by 40mg methylprednisolone daily for 3 days, prednisolone 20mg orally for 1 week. Prednisolone is stopped on day 10 unless patients were on prednisolone prior to transplant, had rejection in the first 10 days or had a primary cause of end stage kidney disease of lupus or vasculitis, where prednisolone is continued at 5mg daily thereafter. |
| --- | --- |
| *Maintenance immunosuppression* | Tacrolimus, started 5 days prior to living donor kidney transplant and on the day of deceased donor kidney transplant targeting trough concentrations of 8-12ng/ml within the first 3 months, 6-8ng/ml from 3-12 months, and 5-7ng/ml thereafter.  Mycophenolate Mofetil (MMF) administered at 2g/day in month 1, 1.5g/day in months 1-12, and 1g/day thereafter. |
| *PCP prophylaxis* | Co-trimoxazole 480mg daily for 3 months |
| *TB prophylaxis* | Isoniazid 300mg daily for 9 months in patients at increased risk of latent tuberculosis (TB) |
| *CMV prophylaxis* | Not given. Valaciclovir is given for 1 month in recipients who are HSV IgG negative. Treatment for CMV is initiated at any level of viremia in seronegative recipients and at 2190 IU/ml in seropositive recipients. |

**Supplementary 2:** Techniques used for HLA antibody detection and HLA typing

| *HLA antibody identification* | HLA antibodies were detected and specificities defined using LABScreen Mixed Screen and Single Antigen beads on the Luminex platform (One Lambda, Thermo Fisher, Scientific, Waltham, MA, USA). HLA antibodies with mean fluorescence intensity (MFI) values greater than 2000 were considered to be positive; cRF was calculated using the National Health Service Blood and Transplant (NHSBT) cRF calculator (<https://www.odt.nhs.uk/transplantation/tools-policies-and-guidance/calculators/>). |
| --- | --- |
| *HLA typing* | High resolution molecular HLA typing to high resolution for 9 loci was performed in 2012-2015 with Luminex LABType (One Lambda, Thermo Fisher, Scientific, Waltham, MA, USA), SBT (in house Sanger sequencing) and SSP (Olerup, Stockholm, Sweden), in 2016-2020 with Luminex LABType, third generation sequencing (in house), and SSP, in 2020-2022 with next generation sequencing (GenDX, Utrecht, The Netherlands) and SSP. |

**Supplementary 3**: Patient Survival (A), allograft survival (B), and death censored allograft survival (C)

stratified by cRF (cRF 0%, cRF 1-97%, and cRF 98-100%).

|  | **Whole population** | **cRF 0%** | **cRF 1-97%** | **cRF 98-100%** | **P-value** |
| --- | --- | --- | --- | --- | --- |
| **Patient Survival** | | | | | |
| 12 months | 1047/1080 (96.94) | 653/669 (97.61) | 353/367 (96.19) | 41/44 (93.18) | >0.99 |
| 36 months | 790/844 (93.60) | 482/514 (93.77) | 283/301 (94.02) | 25/29 (86.21) | >0.99 |
| 60 months | 577/659 (87.56) | 349/400 (87.25) | 214/239 (89.54) | 14/20 (70.00) | 0.34 |
| **Allograft Survival** | | | | | |
| 12 months | 1014/1080 (93.89) | 635/669 (94.92) | 342/367 (93.19) | 37/44 (84.09) | 0.14 |
| 36 months | 746/844 (88.39) | 455/514 (88.52) | 268/301 (89.04) | 23/29 (79.31) | >0.99 |
| 60 months | 516/659 (78.30) | 306/400 (76.50) | 197/239 (82.43) | 13/20 (65.00) | 0.37 |
| **Death Censored Allograft Survival** | | | | | |
| 12 months | 1014/1047 (96.85) | 635/653 (97.24) | 342/353 (96.88) | 37/41 (90.24) | 0.60 |
| 36 months | 746/810 (92.10) | 455/490 (92.86) | 268/291 (92.10) | 23/29 (79.31) | 0.40 |
| 60 months | 516/611 (84.45) | 306/364 (84.07) | 197/228 (86.40) | 13/19 (68.42) | 0.76 |

A.

B.

C.

**Supplementary 4**: Patient Survival (A), allograft survival (B), and death censored allograft survival (C) stratified by cRF (cRF 0%, cRF 1-84%, and cRF 85-100%) in deceased donor kidney transplants.

|  | **Whole population** | **cRF 0%** | **cRF 1-84%** | **cRF 85-100%** | **P-value** |
| --- | --- | --- | --- | --- | --- |
| **Patient Survival** | | | | | |
| 12 months | 772/803 (96.14) | 469/485 (96.70) | 210/217 (96.77) | 93/101 (92.08) | 0.91 |
| 36 months | 568/616 (92.21) | 332/361 (91.97) | 176/186 (94.62) | 60/69 (86.96) | 0.83 |
| 60 months | 415/486 (85.39) | 240/286 (83.92) | 134/148 (90.54) | 41/52 (78.85) | **0.058** |
| **Allograft Survival** | | | | | |
| 12 months | 741/803 (92.28) | 451/485 (92.99) | 204/217 (94.01) | 86/101 (85.15) | 0.16 |
| 36 months | 530/616 (86.04) | 308/361 (85.32) | 167/186 (89.78) | 55/69 (79.71) | 0.099 |
| 60 months | 366/486 (75.31) | 206/286 (72.03) | 123/148 (83.11) | 37/52 (71.15) | **0.026** |
| **Death Censored Allograft Survival** | | | | | |
| 12 months | 741/772 (95.98) | 451/469 (96.16) | 204/210 (97.14) | 86/93 (92.47) | >0.99 |
| 36 months | 530/586 (90.44) | 308/340 (90.59) | 167/180 (92.78) | 55/66 (83.33) | 0.43 |
| 60 months | 366/446 (82.06) | 206/254 (81.10) | 123/142 (86.62) | 37/50 (74.00) | 0.11 |

A.

B.

C.

**Supplementary 5**: Histograms plotting creatinine and eGFR* in the whole population at 12- (a), 36- (b), and 60-months (c).

*eGFR of 90 or greater recorded as 90, and eGFR of 15 or less recorded as 15

a.

b.

c.

**Supplementary 6**: Serum creatinine and eGFR* at 12- (a), 36- (b), and 60-months (c) post-transplant in the entire cohort, and in unsensitised, sensitised and highly sensitised patients

*eGFR of 90 or greater recorded as 90, and eGFR of 15 or less recorded as 15

a.

b.

c.

**Supplementary 7**: Outcomes at 12-, 36-, and 60-months in the whole population and in patients with cRF 0%, cRF 1-97%, and cRF 98-100).

|  | **Whole population** | **cRF 0%** | **cRF 1-97%** | **cRF 98-100%** | **P-value** |
| --- | --- | --- | --- | --- | --- |
| **12-month outcomes** | | | | | |
| Creatinine (μmol/l; median, IQR) | 125 (102-158) | 129 (104-162) | 118 (96-146) | 145 (109-194) | **<0.0001** |
| eGFR (ml/min; median, IQR) | 50 (38-65) | 50 (38-64) | 52 (40-67) | 41 (31-57) | **0.021** |
| Rejection (n; %) | 101/1080 (9.35) | 63/669 (9.42) | 27/367 (7.36) | 11/44 (25.00) | **0.0024** |
| TCMR (n; % of rejection) | 81/101 (80.20) | 56/63 (88.89) | 19/27 (70.37) | 6/11 (54.55) | **0.0088** |
| ABMR (n; % of rejection) | 9/101 (8.91) | 2/63 (3.17) | 4/27 (14.81) | 3/11 (27.27) | **0.012** |
| Mixed / Both TCMR and ABMR (n; % of rejection) | 11/101 (10.89) | 5/63 (7.94) | 4/27 (14.81) | 2/11 (18.18) | 0.35 |
| CMV viremia (n; %) | 259/1080 (23.98) | 154/669 (23.02) | 90/367 (24.52) | 14/44 (31.82) | 0.37 |
| BK viremia (any level) (n; %) | 137/1080 (12.69) | 90/669 (13.45) | 38/367 (10.35) | 9/44 (20.45) | 0.10 |
| BK viremia (>10000 copies/ml) (n; %) | 65/1080 (6.02) | 44/669 (6.58) | 18/367 (4.90) | 3/44 (6.82) | 0.49 |
| **36-month outcomes** | | | | | |
| Creatinine (μmol/l; median, IQR) | 129 (102-175) | 132 (107-179) | 121 (95-167) | 159 (125-234) | **0.0005** |
| eGFR (ml/min; median, IQR) | 47 (33-64) | 47 (32-62) | 48 (35-67) | 36 (26-56) | **0.0289** |
| Rejection (n; %) | 87/844 (10.31) | 50/514 (9.73) | 28/301 (9.30) | 9/29 (31.03) | **0.0047** |
| TCMR (n; % of rejection) | 66/87 (75.86) | 43/50 (86.00) | 18/28 (64.29) | 5/9 (55.56) | **0.027** |
| ABMR (n; % of rejection) | 7/87 (8.05) | 1/50 (2.00) | 3/28 (10.71) | 3/9 (33.33) | **0.0057** |
| Mixed / Both TCMR and ABMR (n; % of rejection) | 14/87 (16.09) | 6/50 (12.00) | 7/28 (25.00) | 1/9 (11.11) | 0.32 |
| Malignancy (n; %) | 52/844 (6.16) | 37/514 (7.20) | 13/301 (4.32) | 2/29 (6.90) | 0.23 |
| Cardiovascular event (n; %) | 34/844 (4.03) | 19/514 (3.70) | 13/301 (4.32) | 2/29 (6.90) | 0.51 |
| **60-month outcomes** | | | | | |
| Creatinine (μmol/l; median, IQR) | 130 (102-187) | 132 (105-192) | 127 (96-172) | 129 (113-250) | 0.08 |
| eGFR (ml/min; median, IQR) | 48 (32-64) | 47 (31-63) | 50 (32-67) | 39 (23-55) | 0.18 |
| Rejection (n; %) | 76/659 (11.53) | 45/400 (11.25) | 25/239 (10.46) | 6/20 (30.00) | **0.047** |
| TCMR (n; % of rejection) | 55/76 (72.37) | 33/45 (73.33) | 19/25 (76.00) | 3/6 (50.00) | 0.48 |
| ABMR (n; % of rejection) | 8/76 (10.53) | 5/45 (11.11) | 1/25 (4.00) | 2/6 (33.33) | 0.112 |
| Mixed / Both TCMR and ABMR (n; % of rejection) | 13/76 (17.11) | 7/45 (15.56) | 5/25 (5.00) | 1/6 (16.67) | 0.89 |
| Malignancy (n; %) | 53/659 (8.04) | 34/400 (8.50) | 17/239 (7.11) | 2/20 (10.00) | 0.70 |
| Cardiovascular event (n; %) | 39/659 (5.91) | 23/400 (5.75) | 14/239 (5.86) | 2/20 (10.00) | 0.59 |

**Supplementary 8**: Clinical characteristics of patients with T cell epitope mismatch data

| Number of patients | 825 |
| --- | --- |
| **Donor Variables** | |
| Age (mean; SD years) | 48.0 (14.5) |
| Sex (n=female; %) | 320 (38.8) |
| Donor type | |
| Live | 221 (26.8) |
| Donor after Brain Death (DBD) | 370 (44.8) |
| Donor after Cardiac Death (DCD) | 234 (28.4) |
| **Recipient variables** | |
| Age (mean; SD years) | 50.0 (13.5) |
| Sex (n=female; %) | 296 (35.9) |
| Ethnicity | |
| White (n; %) | 359 (43.5) |
| Asian (n; %) | 257 (31.2) |
| Black (n; %) | 209 (25.3) |
| Cause of ESKD | |
| Diabetes (n; %) | 191 (23.2) |
| Polycystic kidney (n; %) | 62 (7.5) |
| Pyelonephritis (n; %) | 14 (1.7) |
| Glomerulonephritis (n; %) | 66 (8.0) |
| Other / unknown (n; %) | 492 (59.6) |
| **Transplant variables** | |
| Pre-emptive (n; %) | 186 (22.5) |
| First transplant (n; %) | 701 (85.0) |
| Total HLA-A, -B, -DR Mismatch (mean; SD) | 3.04 (1.35) |
| Total HLA-A, -B, -DR Mismatch 0-3 (n; %) | 538 (65.2) |
| cRF 0% (n; %) | 523 (63.5) |
| cRF 1-84% (n; %) | 214 (26.0) |
| cRF 85-100% (n; %) | 86 (10.5) |

**Supplementary 9**: Cox regression analyses of clinical variables associated with patient survival, graft survival, death-censored graft survival and rejection-free allograft survival. Hazard ratios and 95% confidence intervals are provided for each variable included within the model. cRF and ln(PIRCHE+1) are included as continuous variables.

|  | **PATIENT SURVIVAL** | | **GRAFT SURVIVAL** | | **DEATH-CENSORED GRAFT SURVIVAL** | | **REJECTION-FREE ALLOGRAFT SURVIVAL** | |
| --- | --- | --- | --- | --- | --- | --- | --- | --- |
| ***Clinical Variable*** | ***Hazard ratio*** | ***95% confidence interval*** | ***Hazard ratio*** | ***95% confidence interval*** | ***Hazard ratio*** | ***95% confidence interval*** | ***Hazard ratio*** | ***95% confidence interval*** |
| Age at Transplant | **1.069** | **1.040 to 1.102** | **1.025** | **1.008 to 1.043** | 1.003 | 0.9810 to 1.025 | 0.9883 | 0.9734 to 1.004 |
| Male Sex [reference = female] | 1.112 | 0.5791 to 2.252 | 0.7717 | 0.5010 to 1.202 | **0.5292** | **0.2975 to 0.9415** | **0.6471** | **0.4353 to 0.9653** |
| Ethnicity [black; reference = white] | **0.2844** | **0.09474 to 0.6979** | 0.8054 | 0.4760 to 1.339 | 1.384 | 0.7215 to 2.676 | 1.050 | 0.6598 to 1.660 |
| Ethnicity [Asian; reference = white] | 0.8597 | 0.4259 to 1.676 | 0.7815 | 0.4656 to 1.286 | 0.7516 | 0.3504 to 1.551 | 0.7021 | 0.4180 to 1.151 |
| DBD transplant [reference = live transplant] | 1.495 | 0.5811 to 4.652 | 1.647 | 0.8583 to 3.439 | 1.827 | 0.7593 to 5.137 | 0.8893 | 0.5254 to 1.537 |
| DCD transplant [reference = live transplant] | 0.9511 | 0.3482 to 3.047 | 1.781 | 0.8951 to 3.825 | **2.923** | **1.182 to 8.394** | 1.337 | 0.7783 to 2.342 |
| Total HLA Mismatch | 0.9694 | 0.7359 to 1.274 | 0.8300 | 0.6845 to 1.005 | **0.7678** | **0.5895 to 0.9967** | 0.9290 | 0.7746 to 1.112 |
| cRF as continuous variable | 0.9929 | 0.9787 to 1.006 | 0.9981 | 0.9902 to 1.006 | 1.000 | 0.9908 to 1.009 | 1.002 | 0.9959 to 1.009 |
| Pre-emptive transplant [reference = not pre-emptive] | **0.07112** | **0.003990 to 0.3325** | **0.3336** | **0.1462 to 0.6616** | 0.5434 | 0.2161 to 1.177 | 1.064 | 0.6526 to 1.682 |
| Multiple grafts [reference = first graft] | 2.104 | 0.6491 to 6.017 | 1.227 | 0.5924 to 2.386 | 1.013 | 0.3841 to 2.344 | 1.295 | 0.7247 to 2.220 |
| ln(PIRCHE+1) as continuous variable | 1.170 | 0.7527 to 1.904 | 1.123 | 0.8468 to 1.524 | 1.095 | 0.7661 to 1.626 | **1.324** | **1.012 to 1.779** |

**Supplementary 10**: Cox regression analyses of clinical variables associated with patient survival, graft survival, death-censored graft survival and rejection-free allograft survival. Hazard ratios and 95% confidence intervals are provided for each variable included within the model. cRF and ln(PIRCHE+1) are included as categorical variables.

|  | **PATIENT SURVIVAL** | | **GRAFT SURVIVAL** | | **DEATH-CENSORED GRAFT SURVIVAL** | | **REJECTION-FREE ALLOGRAFT SURVIVAL** | |
| --- | --- | --- | --- | --- | --- | --- | --- | --- |
| ***Clinical Variable*** | ***Hazard ratio*** | ***95% confidence interval*** | ***Hazard ratio*** | ***95% confidence interval*** | ***Hazard ratio*** | ***95% confidence interval*** | ***Hazard ratio*** | ***95% confidence interval*** |
| Age at Transplant | **1.069** | **1.039 to 1.102** | **1.024** | **1.007 to 1.042** | 1.001 | 0.9794 to 1.023 | 0.9873 | 0.9725 to 1.002 |
| Male Sex [reference = female] | 1.201 | 0.6261 to 2.427 | 0.8076 | 0.5227 to 1.261 | **0.5455** | **0.3058 to 0.9720** | **0.6139** | **0.4132 to 0.9150** |
| Ethnicity [black; reference = white] | **0.2972** | **0.09846 to 0.7358** | 0.8140 | 0.4806 to 1.354 | 1.424 | 0.7418 to 2.755 | 1.064 | 0.6693 to 1.682 |
| Ethnicity [Asian; reference = white] | 0.8110 | 0.3981 to 1.598 | 0.7614 | 0.4517 to 1.258 | 0.7733 | 0.3580 to 1.608 | 0.7126 | 0.4231 to 1.171 |
| DBD transplant [reference = live transplant] | 1.619 | 0.6236 to 5.055 | 1.780 | 0.9318 to 3.700 | 1.987 | 0.8337 to 5.531 | 0.9806 | 0.5802 to 1.692 |
| DCD transplant [reference = live transplant] | 1.089 | 0.3978 to 3.488 | 1.967 | 0.9936 to 4.195 | **3.225** | **1.317 to 9.146** | 1.441 | 0.8390 to 2.523 |
| Total HLA Mismatch | 1.041 | 0.7996 to 1.354 | 0.8766 | 0.7278 to 1.054 | 0.7954 | 0.6167 to 1.023 | 0.9791 | 0.8244 to 1.161 |
| cRF 1-84% [reference = cRF 0%) | 0.8784 | 0.4199 to 1.741 | 0.7924 | 0.4729 to 1.289 | 0.7621 | 0.3801 to 1.457 | 0.6876 | 0.4164 to 1.103 |
| cRF 85-100% [reference = cRF 0%) | 0.5911 | 0.1133 to 2.324 | 1.095 | 0.4755 to 2.340 | 1.348 | 0.5074 to 3.259 | 1.546 | 0.8039 to 2.837 |
| Pre-emptive transplant [reference = not pre-emptive] | **0.07016** | **0.003928 to 0.3303** | **0.3413** | **0.1492 to 0.6801** | 0.5796 | 0.2296 to 1.267 | 1.100 | 0.6718 to 1.746 |
| Multiple grafts [reference = first graft] | 1.900 | 0.5881 to 5.297 | 1.083 | 0.5153 to 2.128 | 0.8810 | 0.3273 to 2.079 | 1.275 | 0.7103 to 2.195 |
| ln(PIRCHE+1) quartile 2 [reference = quartile 1] | 0.9276 | 0.4064 to 2.141 | 1.151 | 0.6484 to 2.061 | 1.570 | 0.7269 to 3.524 | 1.398 | 0.7951 to 2.496 |
| ln(PIRCHE+1) quartile 3 [reference = quartile 1] | 1.037 | 0.4090 to 2.606 | 1.123 | 0.5924 to 2.134 | 1.162 | 0.4789 to 2.851 | 1.323 | 0.7068 to 2.493 |
| ln(PIRCHE+1) quartile 4 [reference = quartile 1] | 0.6720 | 0.2420 to 1.795 | 0.8042 | 0.3997 to 1.595 | 1.000 | 0.3840 to 2.580 | 1.557 | 0.8352 to 2.939 |
